# Euclidean distance-optimized data transformation for cluster analysis in biomedical data (EDOtrans)

**DOI:** 10.1186/s12859-022-04769-w

**Published:** 2022-06-16

**Authors:** Alfred Ultsch, Jörn Lötsch

**Affiliations:** 1 DataBionics Research Group, University of Marburg, Hans - Meerwein - Straße, 35032 Marburg, Germany; 2 Institute of Clinical Pharmacology, Goethe - University, Theodor Stern Kai 7, 60590 Frankfurt am Main, Germany; 3 Fraunhofer Institute for Translational Medicine and Pharmacology ITMP, Theodor-Stern-Kai 7, 60596 Frankfurt am Main, Germany

**Keywords:** Data science, Machine-learning, Biomedical informatics, Data preprocessing

## Abstract

**Background:**

Data transformations are commonly used in bioinformatics data processing in the context of data projection and clustering. The most used Euclidean metric is not scale invariant and therefore occasionally inappropriate for complex, e.g., multimodal distributed variables and may negatively affect the results of cluster analysis. Specifically, the squaring function in the definition of the Euclidean distance as the square root of the sum of squared differences between data points has the consequence that the value 1 implicitly defines a limit for distances within clusters versus distances between (inter-) clusters.

**Methods:**

The Euclidean distances within a standard normal distribution (N(0,1)) follow a N(0,$\sqrt{2}$) distribution. The EDO-transformation of a variable X is proposed as $EDO= X/(\sqrt{2}\cdot s)$ following modeling of the standard deviation *s* by a mixture of Gaussians and selecting the dominant modes via item categorization. The method was compared in artificial and biomedical datasets with clustering of untransformed data, z-transformed data, and the recently proposed pooled variable scaling.

**Results:**

A simulation study and applications to known real data examples showed that the proposed EDO scaling method is generally useful. The clustering results in terms of cluster accuracy, adjusted Rand index and Dunn’s index outperformed the classical alternatives. Finally, the EDO transformation was applied to cluster a high-dimensional genomic dataset consisting of gene expression data for multiple samples of breast cancer tissues, and the proposed approach gave better results than classical methods and was compared with pooled variable scaling.

**Conclusions:**

For multivariate procedures of data analysis, it is proposed to use the EDO transformation as a better alternative to the established z-standardization, especially for nontrivially distributed data. The “EDOtrans” R package is available at https://cran.r-project.org/package=EDOtrans.

**Supplementary Information:**

The online version contains supplementary material available at 10.1186/s12859-022-04769-w.

## Introduction

Biomedical data often contain subgroup structures that are identified by data projection and clustering. For this purpose, informatic methods of data projection [1] and cluster identification [2] are available. Clustering is widely used in biomedical research. The quality of clustering plays a critical role in biomedical research and depends on the distance metrics used to quantify similarities and dissimilarities between data points [3], which allows the integration of the different dimensions into a measure of (dis-)similarity. Two problems must be solved for a definition of a valid distance function on the data. First, the dispersions, e.g., the variances respectively standard deviations, of the different variables in the data set must be made comparable. Therefore, standard workflows include data transformation. Second, the design of this transformation must consider the specifics of the distance function used. The importance of similarity metrics on the clustering of biomedical data has been recognized [4]. In many projects, Euclidean distance to z-standardized data is the default approach [5].

A particular problem with Euclidean distance is that it is not scale invariant, i.e., multiplying the data by a common factor changes the distance. Recognizing this potential pitfall in clustering approaches, adapted scaling methods have been proposed that take into account the scale dependence of the Euclidean distance, such as pooled variable scaling (PVS) [6]. In this report, an alternative to the standard z-transform of biomedical data is proposed as a more appropriate approach for clustering biomedical data. The "Euclidean Distance Optimized" (EDO) data transformation addresses the scale dependence of Euclidean distance, but treats each variable separately and therefore does not introduce clustering at the transformation level as alternative approaches do [6]. It specifically takes into account that the squaring function in the definition of Euclidean distance results a breakpoint for distances within (inner)clusters versus distances between (inter)clusters at a value of 1. Thus, while in [6] the goal is to make the scales of different variables comparable, the present approach aims at minimizing differences within classes and maximizing differences between classes. It is therefore more focused on finding clusters in the data. The present work is concerned with numerical data, in particular data variables with an interval scale level of measurement. Fusion of heterogeneous data such as strings or images is not considered. For integration of such data into a single path, see, e.g., [7].

## Methods

### Distributions within and between classes in data sets

Defining a meaningful distance requires expert knowledge, which in many projects is replaced by the "usual procedure" of applying the Euclidean distance to standardized (z-standardized) data [5]. The Euclidean distance is the most intuitive distance metric as it corresponds to the everyday perception of distances. The Euclidean distance *d* of two data cases (x_1_, x_2_) is defined as the square root of the sum of squared differences $d\left(x,y\right)= \sqrt{\sum {\left|{x}_{i}-{y}_{i}\right|}^{2}}$. The Euclidean metric is translation invariant, i.e., it does not change when a common value is added to each variable of the data; however, the Euclidian distance is not scale invariant, i.e., multiplying the data with a common factor changes the distance! That is, using the squaring function (*d*^*2*^) on $d=\left|{x}_{i}-{y}_{i}\right|$ has the effect that differences < 1 become smaller and differences > 1 result in larger values of d^2^. For class or cluster problems, the squaring function in Euclidean distance (see above) has therefore the consequence of implicitly defining 1 as the limit for inner class distances and between (inter) class distances. While this may be appropriate for simple distributions of a variable, such as normal (Gaussian) distributions, it may be inappropriate for more complex variables, such as characteristics with bimodal or multimodal distributions.

**Fig. 1:**
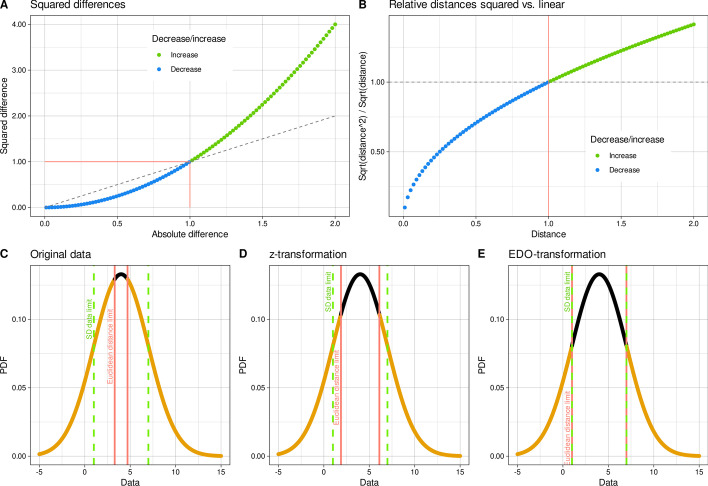
Implicit definition of instances within a class (innerclass instances) in an exemplary distribution using the Euclidean distance. Properties of the Euclidian distance relevant to innerclass and interclass distances. **A** The problem addressed by the EDO transformation has its origin in the behavior of the squared differences function,$f\left(x\right)={x}^{2}$. Here x^2^ < x holds for x values in the interval [1, 1] and x^2^ > x for x values outside this interval, which affects the analogous behavior of the Euclidean distance based on the sum of the squared single differences. The value of x = 1 at which the change occurs is marked by a red solid line. The dashed dark gray lines indicate the identity x^2^ = x. **B** Behavior of Euclidean distances compared to distances computed without using the square of individual distances, again indicating a break from ≤ 1 to > 1 at a distance of $d = 1$ (solid red line). The identity between the two implementations of the distances is shown as a (horizontal) dashed dark gray line. **C–E** Limits on the assignment of a data point to the inner center of a distribution. The green lines mark the distance of one standard deviation from the mean in a normally distributed data set with distribution N(4,3). The red vertical lines mark the boundaries between which a data point has a Euclidean distance ≤ 1 from the center. Data points located within the innerclass rage are colored black, while data points located at greater distances from the center are colored gold. **C** For untransformed raw data, this innerclass range is much narrower than the usual mean ± standard deviation range. **D** When z-standardization is applied, the innerclass range becomes wider. The graph again shows the original data, but the innerclass limits were calculated for z-standardized data and transformed back to the original data range. **E** With the EDO transformation, the innerclass angel finally fulfills the desire to cover the usual mean +—normalization range. Again, the graph shows the original data, but the innerclass limits were calculated for EDO-transformed data and transformed back to the original data range. The figure has been created using the R software package (version 4.1.2 for Linux; https://CRAN.R-project.org/ [9]) and the R libraries “ggplot2” (https://cran.r-project.org/package=ggplot2 [10]) and “ggthemes” (https://cran.r-project.org/package=ggthemes [11])

In data mining and knowledge discovery in multivariate data, the first step is to analyze the distribution of the individual variables. Let $X in {\mathbb{R}}^{d}$ be a multivariate, i.e., d-dimensional data set representing a finite |X|= n number of outcomes (cases) of an experiment. This is typically described as drawn at random from a process that generates the data according to some probability distribution function (*pdf(X)*). Distributions are called simple, if the standard deviation $s\left(x\right)=\frac{1}{n}\sqrt{\sum_{i=1}^{n}{\left(m-{x}_{i}\right)}^{2}}$, for all *x*_*i*_ in *X* with where *m* denotes the arithmetic mean value of in *X*, is an appropriate measure of the dispersion of the variable. Normal or Gaussian distributions are the most encountered types of simple distributions and often serve as standard model. If the values x of a given variable are normally distributed, then an application of the z-standardization *z* = (*x* − *m*)/*s*, where *m* denotes the mean of the values in the variable and *s* the standard deviation, yields a standard normal distribution *z* ~ *N*(0, 1). The Euclidean distances = (*z*_*i*_ − *z*_*j*_) in N(0, 1) follow an N(0, $\sqrt{2}$) distribution [8]. This implicitly defines innerclass distances as distances < 1 and interclass distances as distances > 1 (see the first panel in Fig. 1).

### Algorithm

Considering the distribution parameters, this means that the intraclass versus interclass relevant limit for the distance of a data point from the center is 1$/\sqrt{2}$. Hence, the EDO transformation, provided that a suitable estimate of s is available, is defined as

1 $$EDO= \frac{X}{\sqrt{2}\cdot s}$$

This increases the innerclass range from that for raw data or z-standardized data, i.e., more data points around the arithmetic mean fall within the defined Euclidian distance of < 1 (Fig. 1).

Distributions are called complex if the data generating process produces multimodal distributions that have two or more local maxima (modes, peaks) in their probability distribution function. The reason for such modes may be that the data-generating process operates in different states, e.g., "healthy" versus "sick." Such complex distributions are common in nature and especially in biology. A generative process underlying such multimodal distributed data can be described by a Gaussian mixture model (GMM), which generates data using a sum (i.e., mixture) of conditional probabilities:

2 $$p\left(x\right) = {\sum }_{i = 0}^{M}{w}_{i}N\left(x|{m}_{i},{s}_{i}\right) = {\sum }_{i = 1}^{M}{w}_{i}\cdot \frac{1}{\sqrt{2\pi {s}_{i}}}\cdot {e}^{-\frac{{\left(x-{m}_{i}\right)}^{2}}{{2s}_{i}^{2}}},$$

where *N*(*x|m*_*i*_, *s*_*i*_) (components) denote Gaussian probability densities with means *m*_*i*_ and standard deviations *s*_*i*_. *M* is the number of components in the mixture. The weights *w*_*i*_ denote the relative contribution of each Gaussian component to the overall distribution and add up to a value of 1. The most important or dominant subsets or modes within the data set are those with the largest weights, i.e., the largest prior probability. Which modes belong to this category can be determined using computed ABC analysis [12]. This divides the weights of the Gaussian mixture components into three non-overlapping subsets named “A”, “B”, and “C” [13]. Subset “A” contains the “important few,” i.e., the weights that place the particular Gaussian mode in the “dominant” category. The combined variances within this dominant set of components provide a more adequate measure of the dispersion of the data than the z-standardization. This will be discussed in more detail later in this report. When there is no dominant mode, e.g., when all classes are equally weighted, the median of the standard deviations of the modes in ABC set “B” is used in the EDO transformation.

The standard deviation relevant for the EDO transformation is that of the dominant modes. In the case where more than one mode has been assigned to the dominant category, the combined standard deviation ${s}_{combined}$ is calculated as

3 $${s}_{combined}=\sqrt{\frac{(\sum_{i=1}^{M}\left(\left({n}_{i}-1\right)\cdot {s}_{i}^{2}+{n}_{i\cdot }\cdot {{m}_{i}}^{2}\right)-n\cdot {{m}_{w}}^{2}}{n-1}}$$

where ${n}_{i}={w}_{i}\cdot n$ is the relative number of data in mode *i* and ${m}_{w} = \sum_{1}^{M}\left({w}_{i}\cdot {m}_{i}\right)$ the weighted mean in the GMM. The EDO transformation on multimodally distributed one-dimensional data is then defined as

4 $$EDO= \frac{X}{\sqrt{2}\cdot {s}_{combined}}$$

**Fig. 2:**
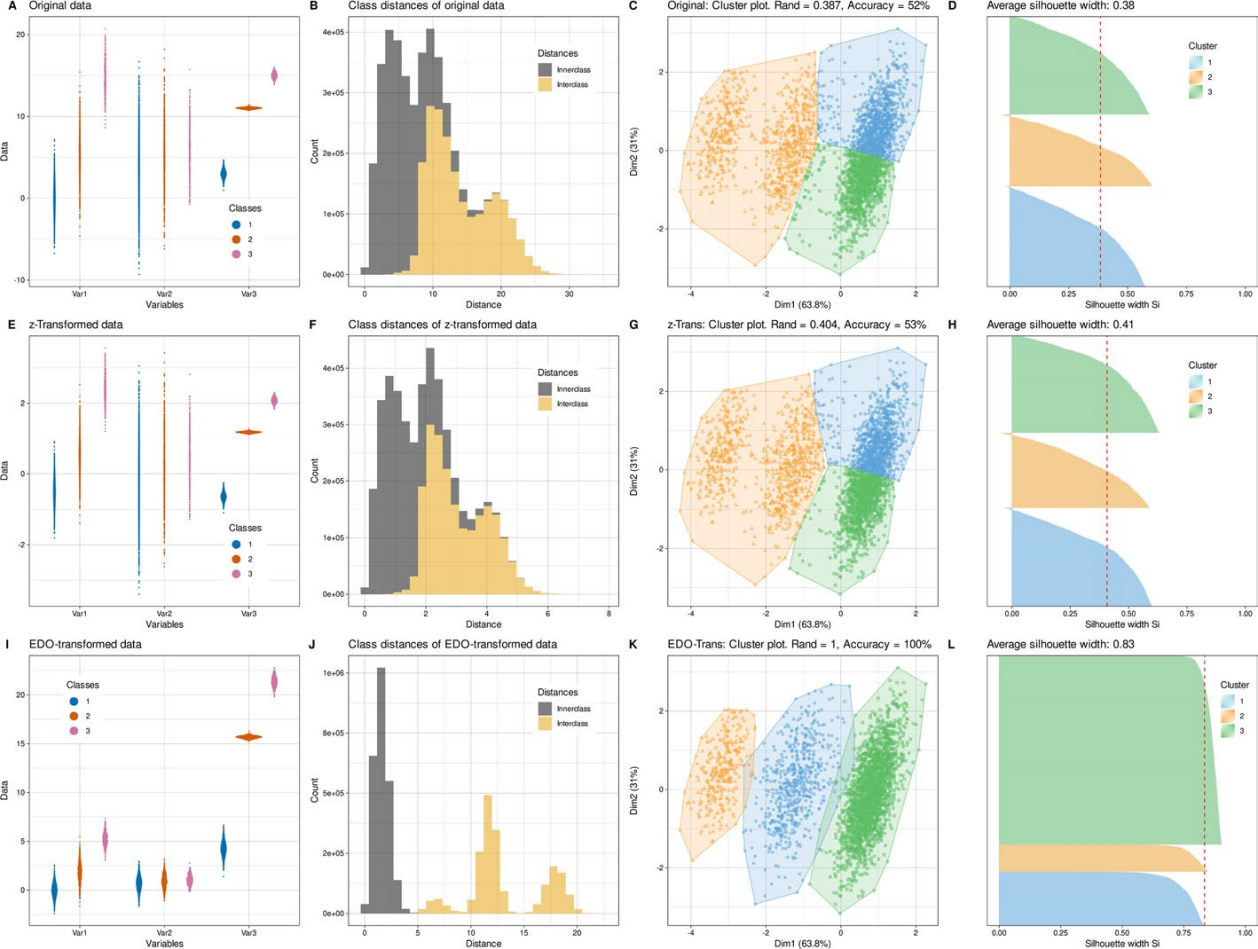
Effects of EDO transformation on innerclass and interclass distances and clustering of multivariate datasets. K-means clustering of an artificial data set that represented a three-class scenario with values generated by Gaussian mixture models with four different variables with increasing means, various standard deviations with a total of 3000 instances with class weights = [0.7, 0.2, 0.1] in each variable. The clustering was performed on untransformed (raw) data (panels **A**–**D**), on z-standardized data (panels **E**–**H**), and on EDO transformed data (panels **I**–**L**). For each kind of data transformation, four panels are shown. The left panels **A**, **E**, **I** show the original data that consist of three variables that are distributed according to a Gaussian mixture containing three modes. The sinaplot [15] shows the individual data points of the three subgroups dithering along the x-axis to create a contour indicating the probability density of the distribution of the data points. Panels **B**, **F**, **J** show the distribution of innerclass and interclass distances as histograms. Panels **C**, **G**, **H** show factorial plots of the individual data points on a principal component analysis projection colored according to a k-means clustering. The borders of the colored areas visualize the cluster separation. The right panels **D**, **H**, **L** show Silhouette plots for the three clusters. Positive values indicate that the sample is within a cluster while negative values indicate that those samples might have been assigned to the wrong cluster because they are closer to neighboring than to their own cluster. The figure has been created using the R software package (version 4.1.2 for Linux; https://CRAN.R-project.org/ [9]) and the R packages “ggplot2” (https://cran.r-project.org/package=ggplot2 [10]), and “FactoMineR” (https://cran.r-project.org/package=FactoMineR [16]). The colors were selected from the “colorblind_pal” palette provided with the R library “ggthemes” (https://cran.r-project.org/package=ggthemes [11])

It is often known that the data is generated by a data generating process that operates in different states (e.g., healthy or sick), i.e., a prior classification of the data is given. The EDO transformation could then be calculated using this prior classification. The distribution of the distances of the data points can be divided into innerclass and interclass according to the previous classification: If two cases *x* and *y* are from the same prior class, the distance is an innerclass distance, otherwise *x* and *y* are from two different classes and therefore their distance is classified as interclass distance. Based on these considerations, applying the EDO transformation to a three-class data set with three variables distributed according to Gaussian mixtures with M = 3 modes resulted in the expected improvement in k-means based clustering [14] (Fig. 2).

However, the underlying assumption that the pre-classification structure is reflected in the Euclidean distance structures may not always be valid. Assume a measured variable *X* that after statistical testing can be assumed to be normal Gaussian distributed. Let the pre-classification into healthy versus sick be such that if *x* ≤ *mean*(*X*), the diagnosis is that *x* is healthy, if *x* > *mean*(*X*), the diagnosis is that *x* is sick. In this type of pre-classification, the distance structures between healthy and sick are indistinguishable. Therefore, it is advisable to base the EDO transformation on the observed modes of the distribution of the variable rather than on the pre-classification structure.

### Experimentation

The programming work for this report was performed in the R language [17] using the R software package [9] (version 4.1.2 for Linux), which is available free of charge in the Comprehensive R Archive Network at https://CRAN.R-project.org/. Considering the goal of EDO data transformation to improve subgroup separation, e.g., clustering, the experiments were performed with artificial datasets created to have the required subgroup structure, or with biomedical data for which a subgroup structure was known. Clustering results were compared between the use of raw data, standard z-transformed data, and EDO-transformed data. The EDO transformation was performed following the analysis of each variable for the modal distribution using the automated Gaussian mixture modeling implemented in the R library “opGMMassessment” (https://cran.r-project.org/package=opGMMassessment).

Partitioning based clustering was mainly implemented as k-means clustering [14]; however, partitioning around medoids (PAM) was used for comparison [18]. Hierarchical clustering with Ward's linkage [19] was used; however, average and complete linkage were used for comparison in analogy to the choice made in [6]. The Euclidean distance was used as the target of the transformation method presented here. Clustering was done using the R package “cluster” (https://cran.r-project.org/package=cluster [20]). Cluster quality and stability were assessed by calculating the cluster accuracy and the adjusted Rand index [21] against the prior classification of the data, and as Dunn’s index [22], calculated using the R packages “fossil” (https://cran.r-project.org/package=fossil [23]) and “clValid” (https://cran.r-project.org/package=clValid [24]). Clustering was compared to a modern alternative scaling approach targeting Euclidean distance boundaries, recently proposed as pooled variable scaling (PVS) [6], in which, unlike the present method, scaling assumes a k-means clustering analysis of the entire dataset.

### Implementation

The EDO data transformation method proposed here has been implemented in the R package “EDOtrans”, which is available at https://cran.r-project.org/package=EDOtrans. The transformation process of a one-dimensional variable can be called with the function "EDOtrans(Data, Cls, PlotIt = FALSE, FitAlg = "normalmixEM", Criterion = "LR", MaxModes = 8, MaxCores = getOption("mc.cores", 2L), Seed)". At least one data vector ("Data") is expected as input (1). If available, class information (2) per instance ("Cls") can be entered, which is then used as the basis for EDO transformation. The class information can be the prior classification as used in the proof-of-concept in this report, or it can be obtained in any way, e.g., through interactive Gaussian mixture modeling with the R library "AdaptGauss" (https://cran.r-project.org/package=AdaptGauss [25]), which allows data analysis under visual control which may capture the entire modal structure in more complicated cases better than fully automated solutions. However, as used in the experiments in this report, the class information can be omitted and then be created internally in the “EDOtrans” library, using Gaussian mixture modeling imported from the R package “opGMMassessment” (https://cran.r-project.org/package=opGMMassessment). In this case, subsequent parameters such as the fitting algorithm, "FitAlg", the criterion for determining the number of modes in the mixture, "Criterion", and the maximum number of modes, “MaxModes”, are forwarded to that library. More detailed hyperparameter settings are beyond the scope of this report and are provided via the R library help function.

## Results

### Proof of concept study

A three-dimensional data set was created with 3000 instances drawn from three normal distributions with different probabilities, resulting in three-modal data (M = 3 modes). For the three variables of which each followed a three-modal distribution, the class weights were always w_i_ = [0.7, 0.2, 0.1] for classes 1 to 3. However, the means and standard deviations differed for the Gaussian mixtures, with parameter values for mixture no. 1 were means = [0, 5, 15] and standard deviations = [2, 2, 3], for mixture no. 2 were means = [4, 5, 6] and standard deviations = [4, 4, 3], and for mixture no. 3 were means = [3, 11, 15] and standard deviations = [0.5, 0.1, 0.4].

For this data set, standard clustering with k-means failed on the raw data, although at least variables #1 and #3 appeared to break into three groups (Fig. 2 top row of panels). The distribution of distances separately by class membership showed that there was a large overlap between within-class and between-class distances. This was little changed by z-standardization, which improved clustering only slightly by 1% accuracy (Fig. 2 middle row of panels). The EDO transformation was performed based on the previous classification. After the EDO transformation of each variable, the clustering solution appeared almost perfect in this sample data set (Fig. 2 bottom row of panels). Replacing k-means with PAM clustering did not change these observations (Additional file 1: Fig. S1).

### Simulation study

A four-dimensional data set with 1,000 instances drawn from three normal distributions each with different probabilities resulting in a three-modal distribution (M = 3 modes) in a Gaussian mixture model (GMM). Each of the three normal distributions is characterized by its expected value *m*_*i*_ and standard deviation *s*_*i*_. The probability that an event is drawn from a certain normal distribution is described by a weighting parameter *w*_*i*_ so that $\sum_{i=1}^{M}{w}_{i}=1$. Specifically, parameters *m*_*i*_, *s*_*i*_, and *w*_*i*_, of the Gaussian mixtures M1, …, M4 were M1: m_i_ = [− 9, − 3, 10], s_i_ = [3, 4, 5], w_i_ = [0.12, 0.05, 0.83], M2: m_i_ = [− 2, 0, 5], s_i_ = [2, 4, 2], w_i_ = [0.39, 0.48, 0.13], M3: m_i_ = [− 2, 0, 5], s_i_ = [2, 4, 2], w_i_ = [0.39, 0.48, 0.13], and M4: m_i_ = [− 6, 0, 2], s_i_ = [3, 1, 5], w_i_ = [0.27, 0.06, 0.67]. This data set is available in the R library "EDOtrans" as “GMMartificialData”. Seven classes were obtained via combining the GMM decisions for all individual variables.

**Fig. 3:**
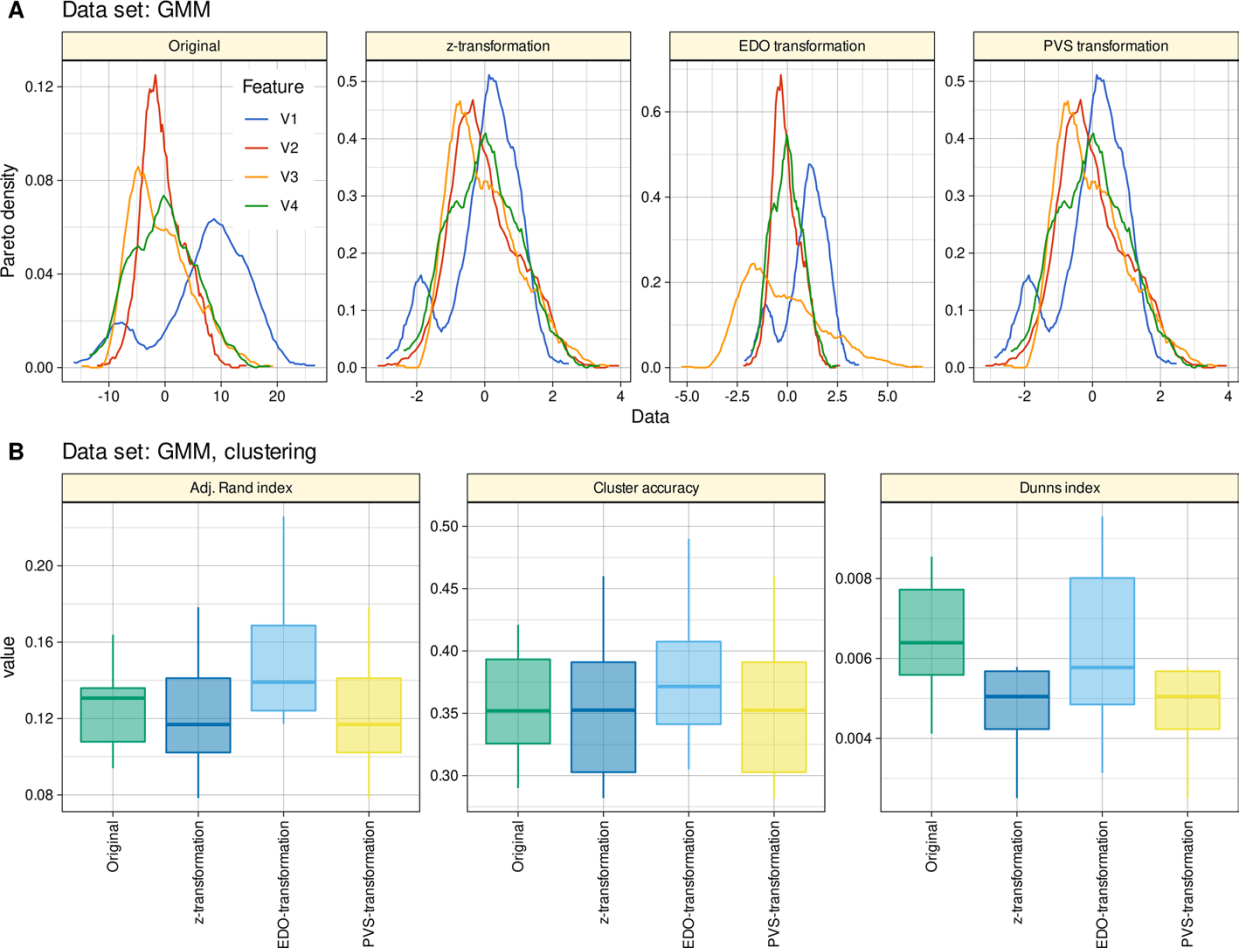
Results of hierarchical cluster analysis of a four-dimensional data set with 1,000 instances created from Gaussian mixtures with M = 3 modes (“GMMartificialData”). **A** The original and transformed data (z-transformation, EDO transformation, and PVS transformation [6]) are shown as a probability density function (PDF) estimated using the Pareto density estimation (PDE [27]), which was developed as a nonparametric kernel density estimator to improve subgroup separation in mixtures. **B** Cluster quality and stability assessed a as cluster accuracy and adjusted Rand index [21] against the prior classification of the data, and as Dunn’s index [22]. The boxes were constructed using minimum, quartiles, median (solid line inside the box) and maximum. The whiskers add 1.5 times the inter-quartile range (IQR) to the 75th percentile or subtract 1.5 times the IQR from the 25th percentile. The figure has been created using the R software package (version 4.1.2 for Linux; https://CRAN.R-project.org/ [9]) and the R packages “ggplot2” (https://cran.r-project.org/package=ggplot2 [10]), and “FactoMineR” (https://cran.r-project.org/package=FactoMineR [16]). The colors were selected from the “colorblind_pal” palette provided with the R library “ggthemes” (https://cran.r-project.org/package=ggthemes [11])

In a tenfold cross-validation scenario, random samples of n = 1000 instances were drawn from the original dataset with replacement by bootstrap resampling [26]. Each variable (Fig. 3 A) was the used untransformed, z-transformed, EDO-transformed after automatic detection of the number of modes using the R package "EDOtrans" described above, and PVS-transformed using the R script provided with the original publication of this method [6]. Internally, this uses majority voting among several methods to determine the number of modes, was imported from the R package "NbClust" (https://cran.r-project.org/package=NbClust), and then performs standard k-means clustering with 100 initial random seeds to determine the center of the initial clusters, and the results are then used to perform the PVS transformation.

The results of hierarchical clustering using Ward's method showed that the clustering compared to the prior classification was best when EDO-transformed variables were used (Fig. 3B). For this dataset, the z-transformation and the PVS-transformation gave the poorest results. However, in the bootstrap scenario, the clustering solutions were generally modest, as indicated by the relatively low values of cluster accuracy and the Rand and Dunn’s indices. Replacing Ward's linkage with average or complete linkage did not change the results in terms of the relative impact of the data transformation methods used on the cluster quality (see Additional file 1: Figs. S1–S7).

### Application of EDO transformation for clustering of further artificial and real datasets

#### Artificial data example

**Fig. 4:**
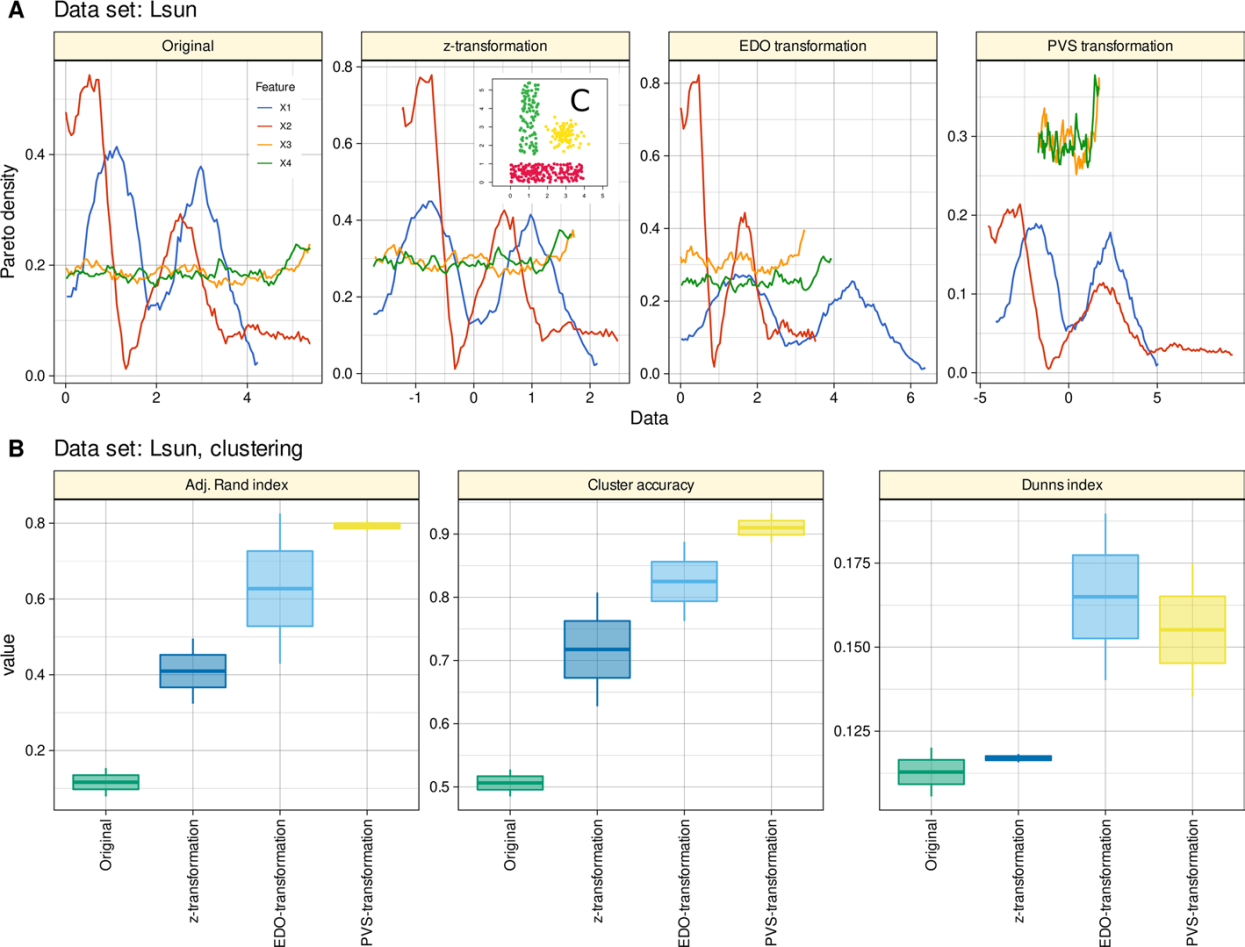
Results of hierarchical cluster analysis of a modified version of the “Lsun” dataset form the “Fundamental Clustering and Projection Suite” (FCPS) [28]. The data set n = 400 instances with d = 4 variables (X1–X4), of which 2 variables are original and two were the same variables but randomly permuted, and k = 3 classes (see insert **C**). **A** The original and transformed data (z-transformation, EDO transformation, and PVS transformation [6]) are shown as a probability density function (PDF) estimated using the Pareto density estimation (PDE [27]), which was developed as a nonparametric kernel density estimator to improve subgroup separation in mixtures. **B** Cluster quality and stability assessed a as cluster accuracy and adjusted Rand index [21] against the prior classification of the data, and as Dunn’s index [22]. The boxes were constructed using minimum, quartiles, median (solid line inside the box) and maximum. The whiskers add 1.5 times the inter-quartile range (IQR) to the 75th percentile or subtract 1.5 times the IQR from the 25th percentile. The figure has been created using the R software package (version 4.1.2 for Linux; https://CRAN.R-project.org/ [9]) and the R packages “ggplot2” (https://cran.r-project.org/package=ggplot2 [10]), and “FactoMineR” (https://cran.r-project.org/package=FactoMineR [16]). The colors were selected from the “colorblind_pal” palette provided with the R library “ggthemes” (https://cran.r-project.org/package=ggthemes [11])

The “Lsun” dataset belongs to the so-called “Fundamental Clustering and Projection Suite” (FCPS), occasionally also referred to as “Fundamental Clustering Problems Suite”, of which the most comprehensive description has been published in [28]. The data set consists of three well-separated data classes, but with different convex hulls: a sphere and two "bricks" of different size (insert C in Fig. 4). This structural property raises the problem of different variances or densities in the cluster. The original data set consists of n = 400 instances with d = 2 variables and k = 3 classes. To make the task slightly more difficult, in the present experiments the variables were included twice, one in the original version and again after random permutation.

The results of hierarchical clustering using Ward's method showed that the best cluster accuracy and adjusted Rand index when the PVS-transformation was used, while the best Dunn’s index provided the EDO transformation (Fig. 4). Both innovative methods outperformed the classical methods of z-transformation or using untransformed variables.

#### Flow cytometric data example

**Fig. 5:**
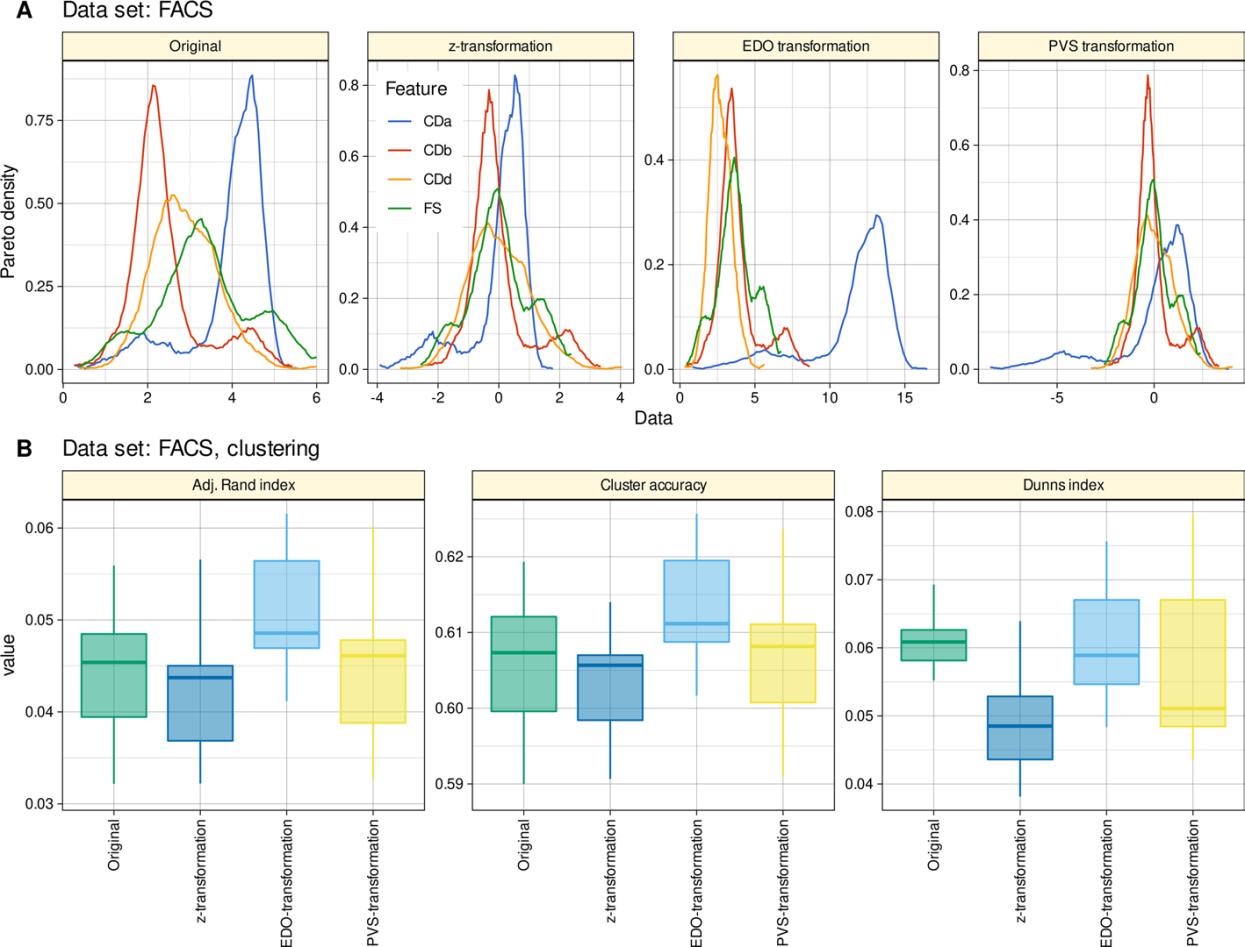
Results of hierarchical cluster analysis of a four-dimensional data set with 3,000 instances of flow cytometric (FACS) measurements modes (“FACSData”). **A** The original and transformed data (z-transformation, EDO transformation, and PVS transformation [6]) are shown as a probability density function (PDF) estimated using the Pareto density estimation (PDE [27]), which was developed as a nonparametric kernel density estimator to improve subgroup separation in mixtures. **B** Cluster quality and stability assessed a as cluster accuracy and adjusted Rand index [21] against the prior classification of the data, and as Dunn’s index [22]. The boxes were constructed using minimum, quartiles, median (solid line inside the box) and maximum. The whiskers add 1.5 times the inter-quartile range (IQR) to the 75th percentile or subtract 1.5 times the IQR from the 25th percentile. The figure has been created using the R software package (version 4.1.2 for Linux; https://CRAN.R-project.org/ [9]) and the R packages “ggplot2” (https://cran.r-project.org/package=ggplot2 [10]), and “FactoMineR” (https://cran.r-project.org/package=FactoMineR [16]). The colors were selected from the “colorblind_pal” palette provided with the R library “ggthemes” (https://cran.r-project.org/package=ggthemes [11])

Biomedical empirical data from flow cytometry using fluorescence-activated cell sorting (FACS) were available from a hematologic data set. For the present experiments, d = 4 variables including the value of the forward scatter (FS) and cytological makers (CD) called for nondisclosure reasons a, b and d, which were downsampled from originally n = 111,686 cells obtained from 100 patients with chronic lymphocytic leukemia and 100 healthy control subjects to n = 3,000 instances. This data set (Fig. 5A) is available in the R library "EDOtrans" as “FACSdata” and consist of a subsample of a larger data set published at https://data.mendeley.com/datasets/jk4dt6wprv/1, (accessed March 1, 2022) [29]. The original study followed the Declaration of Helsinki and was approved by the Ethics Committee of Medical Faculty of the Phillips University of Marburg, Germany.

The results of hierarchical clustering using Ward's method showed that the clustering compared to the prior classification was again best when EDO-transformed variables were used (Fig. 5B). Z-transforming the individual variables resulted in poorer clustering results in terms of accuracy and Rand or Dunn’s indices than using the original, untransformed variables, while PVS-transforming resulted in clustering results comparable to those obtained with the untransformed dataset.

#### Iris flower data example

The Iris flower data set was included for its wide use in statistics for testing of methods and because it was also used for the introductory simulation study in the report on the PVS method, which serves here as a comparative method [6]. The Iris data set gives the measurements in centimeters of the four variables sepal length and width or petal length and width for 50 flowers each of the three species *Iris setosa*, *versicolor* and *virginica*. As there are apparently at least half a dozen different versions of this data set, it is necessary to specify that in the present analysis, the version implemented in R software package as “data(iris)” was used. Table 1 illustrates the effect of scaling with the proposed EDO transformation on this data and compares it with the SD and the range.

**Table 1:**
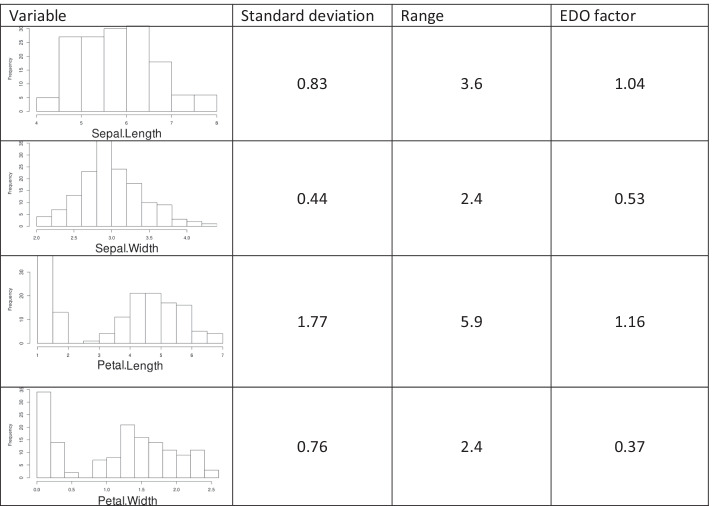
The effect of variable scaling on the Iris flower data set

Compare Table 1 in [6]

**Fig. 6:**
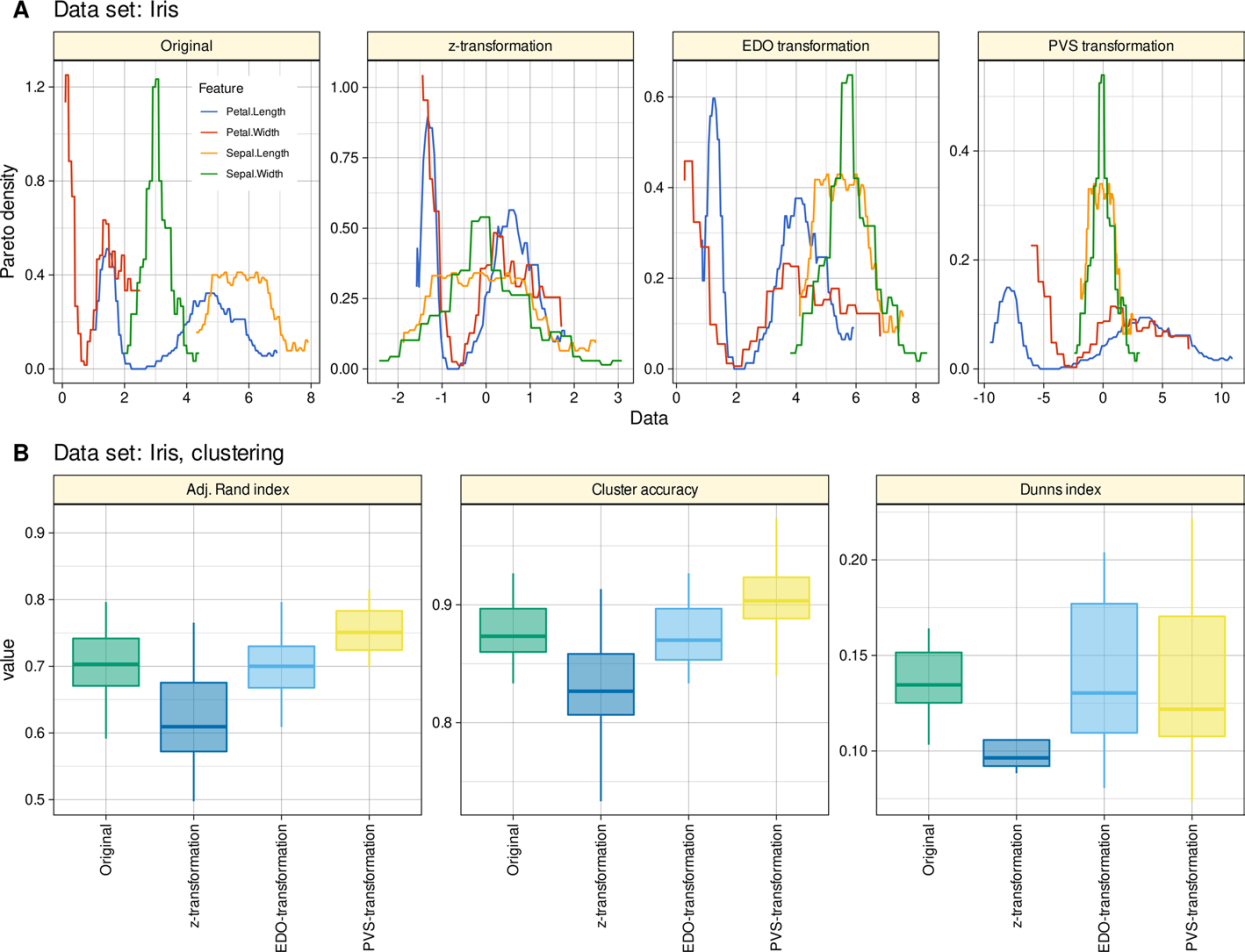
Results of hierarchical cluster analysis of a four-dimensional data set with 150 instances of three species of Iris flower [30, 31] (“Iris”). **A** The original and transformed data (z-transformation, EDO transformation, and PVS transformation [6]) are shown as a probability density function (PDF) estimated using the Pareto density estimation (PDE [27]), which was developed as a nonparametric kernel density estimator to improve subgroup separation in mixtures. **B** Cluster quality and stability assessed a as cluster accuracy, adjusted Rand index [21] against the prior classification of the data, and as Dunn’s index [22]. The boxes were constructed using minimum, quartiles, median (solid line inside the box) and maximum. The whiskers add 1.5 times the inter-quartile range (IQR) to the 75th percentile or subtract 1.5 times the IQR from the 25th percentile. The figure has been created using the R software package (version 4.1.2 for Linux; https://CRAN.R-project.org/ [9]) and the R packages “ggplot2” (https://cran.r-project.org/package=ggplot2 [10]), and “FactoMineR” (https://cran.r-project.org/package=FactoMineR [16]). The colors were selected from the “colorblind_pal” palette provided with the R library “ggthemes” (https://cran.r-project.org/package=ggthemes [11])

The results of hierarchical clustering using Ward's method (Fig. 6) resulted in an inverse ranking between EDO and PVS transformations as observed for the FACS dataset. The PVS method provided the best preprocessing when judged by cluster accuracy, the Rand index calculated against the prior classification and Dunn’s index. Nevertheless, both the EDO and PVS transforms outperformed the classical approaches, especially the z-transform before clustering.

#### Gene expression example

**Fig. 7:**
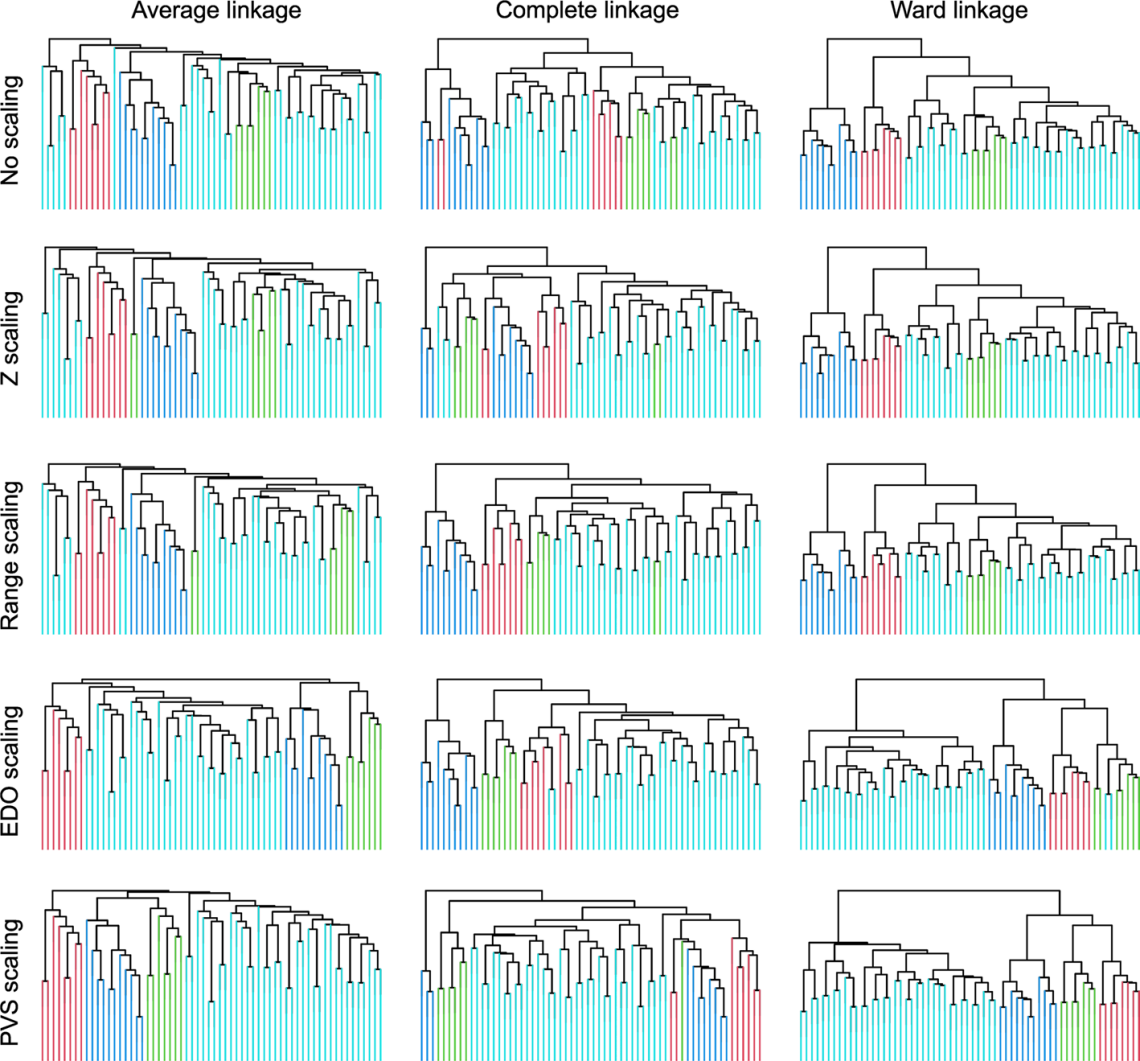
The effect of variable scaling on the gene expression data. The data set comprised 65 surgical samples of human breast tumors in which hierarchical clustering of the expression of 496 intrinsic genes that showed significantly greater variation between different tumors than variation between paired samples of the same tumor had resulted in four distinct tumor types [32]. The dendrogram colors correspond to the tumor type: basal-like in red, Erb-B2þ in green, normal-breast-like in dark blue and luminal epithelial/ERþ in cyan. The EDO transformation generally yields superior recovery of the true clusters, comparable with the PVS transformation. The experiment is a re-run of the experiment performed for Fig. 3 in [6], using the R script available at https://wis.kuleuven.be/statdatascience/robust/Programs/pooledVariableScaling/pvs-r.zip with addition of code implementing the present EDO transformation

Another set of biomedical empirical data were the gene expression patterns of 65 surgical samples of human breast tumors provided in the supplementary materials of [6]. The data originate from a publication of patterns in 496 intrinsic genes that showed significantly greater variation between different tumors than variation between paired samples of the same tumor, resulting in four distinct tumor types by applying hierarchical clustering, including (1) ER+/luminal-like, (2) basal-like, (3) hereditary B2+, and (4) normal breast [32]. The dataset was used in a replication of the experiment conducted by Raymaekers and Zamar [6], using their R script available at https://wis.kuleuven.be/statdatascience/robust/Programs/pooledVariableScaling/pvs-r.zip to compare the effects of different data transformations on the results of hierarchical clustering with average, complete, and Ward linkage. Figure 7 shows the resulting dendrograms when applying each of these clustering algorithms to the dataset after different scaling. For average linkage, EDO scaling outperformed classical transformations or non-transformations, and its results were at the same level as PVS scaling. For complete linkage, EDO scaling misclassified three observations, PVS scaling was wrong for five observations, while range scaling yielded only two errors. This was reversed for Ward clustering, as the EDO transformation produced two more errors than the PVS, but both outperformed the other options.

Using the clustering algorithms chosen by [6] for the previous datasets (Gaussian mixture, "Lsun," FACS data, and iris flower data) also revealed a heterogeneous picture, although a tendency for clustering to improve over classical methods also prevailed for average and complete linkage when all three measures of cluster quality, i.e., cluster accuracy, Rand index, and Dunn’s index, were considered (see Additional file 1: Figures). It should be noted, however, that average and complete linkage seemed to benefit less from the EDO or PVS transformation than Ward’s linkage.

## Discussion

The present experiments have shown that a data transformation that takes into account the inherent properties of the Euclidean distance metric by addressing the inflection point at 1 from distance decreasing to distance increasing effects, as well as the N(0,$\sqrt{2}$) distribution of innerclass distances, can improve the clustering of multidimensional data. Since Euclidean distance is by far the most used distance metric for data projection and subgroup assessment, it is virtually the standard implemented by default in statistical software and rarely specifically mentioned in scientific reports. Therefore, the present proposal of an improved data transformation adapted to this distance metric is relevant for the analysis of biomedical or other data sets.

All non-trivial analyses of multivariate (high-dimensional) data require a distance function (metric) to allow the comparison of cases. There are more than a thousand different distance functions besides the Euclidean metric (for an overview, see [33]). Selecting a suitable distance function is crucial for visualizing the data, i.e., projecting the high-dimensional data into two or three dimensions, and for identifying subgroups or clusters in the data (clustering). Clustering aims to group the cases into a finite number of clusters, in such a way that the objects in one cluster are more similar to each other and more dissimilar to the cases in other clusters. Similarities and dissimilarities are determined by the distance function. The selection of a meaningful and appropriate distance function is therefore the key issue in the analysis of complex multivariate data. Unfortunately, there are few theoretical properties that can be used to identify an appropriate distance function. Moreover, if too many seemingly simple properties such as "identity of indistinguishable data" and "scale invariance" are required of the metric, it can even be shown that subsequent data analysis such as clustering is impossible [34].

However, some requirements for a distance function are essential. The first is translation invariance. That is, it should not matter where the origin of the high-dimensional data space lies. This is equivalent to the requirement that the addition or subtraction of a constant to any of the dimensions of the data should not change the (dis-)similarities within the data. For metric distances, three axioms must be satisfied for this to happen: Identity of indiscernibles (= non-negativity), symmetry, and the triangle inequality. The postulation of segment additivity [35] reduces the admissible distance functions d(x,y) between data points *x* and *y* to Minkowski distances [36]. These have the general formula:

$${d\left(x,y\right)}_{m}={\left(\sum_{i=1}^{d}{\left|{x}_{i}-{y}_{i}\right|}^{m}\right)}^\frac{1}{m}$$

Among these Minkowski distances, the Euclidean distance, with m = 2, is the only metric invariant to orthogonal rotations of the coordinate system. Moreover, the Euclidean distance is experienced in the everyday 3-dimensional world. However, it is often ignored that Euclidean distance has a property that is critical to the success or failure of clustering: it is not invariant to the scaling of the data. As explained above, this means that the similarity or dissimilarity of cases in the data depends on whether the Euclidean distance was applied to the data in its original form or to variables that were transformed (scaled), even if a common scaling factor was used for all variables.

Furthermore, for any comparison of different dimensions, the dispersion, also called scatter or variance, of the variables is important, i.e., a measure of how different the data are within the respective dimension. A key issue in choosing an appropriate distance function is to make a rational choice of this comparison between the different scatters. The standard choice for this is to z-standardize the data. The implicit assumption in using the z-standardization as a scaling prior to applying the Euclidean distance is that the standard deviation (variance) is an appropriate description of the dispersion of the variables. This is valid for simple distributions, i.e., Gaussian-like distributions. However, it encounters problems when the data are less simply distributed, such as multimodal data. For example, an 80/20 (weights = [0.8, 0.2]) bimodally distributed variable with means = [− 5, 5] and small standard deviations of 0.5 for both modes indicates two separate groups that are in themselves quite homogeneous. However, their joint standard division is 4.0 compared to 0.5 for the two separate modes. Therefore, the z-standardization uses a large span, resulting in low values that may become relevant in subsequent projections of the data, considering the scale sensitivity of the Euclidean distance metric. Here, the EDO transformation, by using only the dispersion of the dominant variable, i.e., a standard deviation of 0.5 multiplied with the value of $\sqrt{2}$, leads to larger transformed data, which has consequences for the subsequent application of Euclidean distances in projecting and clustering procedures.

It should be noted that for empirical data with skewed distributions such as exponential or lognormal distributions, it is recommended to transform the data towards normality before applying the EDO algorithm. An example of such a transformation is the Box-Cox transformation [37]. Such a transformation also often eliminates outliers in the original distributions. The EDO transformation is likely to be susceptible to outliers. Treatment of outliers should also be done before applying the EDO method or any other range transformation. To keep this paper short and sweet, we assume that skewed distributions and outliers are part of "pre-processing" before applying a transformation such as the Z or EDO transformation.

The presently proposed EDO transform can be considered as an alternative to the also recently proposed PVS transformation [6], which was developed with a similar goal of adapting the data transformation during preprocessing for clustering to the scale dependence of the Euclidean distance. Neither transformation was always ranked first in the present cluster experiments, while in most cases of the present experimentation both methods were superior to using untransformed data or z-standardization as a preprocessing approach. However, the PVS and EDO transforms differ in their underlying theoretical considerations. That is, PVS assumes that the k-means algorithm yields a valid clustering of the high-dimensional data. This is equivalent to assuming that all such clusters are in the form of hyperspheres and that their decision boundaries are hyperplanes [38]. K-means is a gradient descent algorithm and is therefore sensitive to the specification of points and any local minimal solutions. In practical situations, it is impossible to confirm or falsify this model assumption. EDO exploits the often-overlooked fundamental property of Euclidean distances d within (d < 1) and between classes (d > 1). For high-dimensional data, it is assumed that a valid classification of the data can be modeled as independent Bayesian models using Gaussian mixtures (GMM) [39]. The resulting model for the decision boundaries of the high-dimensional classes are conic sections. Algorithms for fitting the GMM in each dimension, such as expectation maximization, are typically also gradient descent methods but their results can be confirmed or falsified for each variable.

## Conclusions

The Euclidean distance has a peculiarity that is well known but less considered in practice. Namely, the Euclidean distance is not invariant to the scaling of the variables of the data set. The reason for this is the squaring of the differences of the data when calculating the Euclidean distance. The squaring function has the property that it changes its behavior exactly at the value 1: Differences smaller than 1 are reduced, differences larger than 1 are overemphasized quadratically. The EDO transformation takes advantage of this particularity: The scaling of the data is adjusted to map differences of data points within the same group (mode, clustering) to a range smaller than 1. EDO itself is neither a distance nor a clustering algorithm, but is intended as a more reasonable transformation than, for example usual standardization (Z-transformation). The EDO transformation is especially recommended when the Euclidean distance is used in later steps of multivariate data analysis, such as projections, visualizations, or clustering.

## Supplementary Information

**Additional file 1.** Supplemental figures, showing the results of the proof-of-concept study using PAM clustering instead of k-means, and results of the three experiments with the data sets of Gaussian mixtures, Iris flowers and FACS data, using average or complete linkage instead of Ward’s linkage.

## Data Availability

The “EDOtrans” R package is freely available at https://cran.r-project.org/package=EDOtrans. It contains the data sets used in this report and not elsewhere available as referenced. Results of the proof-of-concept study using PAM clustering instead of k-means, and results of the four experiments with the data sets of Gaussian mixtures, Iris flowers, FACS data and “Lsun”, using average or complete linkage instead of Ward’s linkage, are provided as Additional file 1: Figures.
