# Supplementary material for: Euclidean distance-optimized data transformation for cluster analysis in biomedical data (EDOtrans)
Source: BMC Bioinformatics. 2022 Jun 16;23:233. doi: 10.1186/s12859-022-04769-w (PMC9202178; doi:10.1186/s12859-022-04769-w)

# Supplementary Figures

## Partitioning around medoids (PAM)

Supplementary Figure 1: **Effects of EDO transformation on innerclass and interclass distances and clustering of multivariate datasets (analogous to Figure 2 in the main report).** PAM clustering of an artificial data set that represented a three-class scenario with values generated by Gaussian mixture models with four different variables with increasing means, various standard deviations with a total of 3,000 instances with class weights = [0.7, 0.2, 0.1] in each variable. The clustering was performed on untransformed (raw) data (panels A – D), on z-standardized data (panels E – H), and on EDO transformed data (panels I – L). For each kind of data transformation, four panels are shown. The left panels **A, E, and I** show the original data that consist of three variables that are distributed according to a Gaussian mixture containing three modes. The sinaplot shows the individual data points of the three subgroups dithering along the x-axis to create a contour indicating the probability density of the distribution of the data points. Panels **B, F, and J** show the distribution of innerclass and interclass distances as histograms. Panels **C, G and H** show factorial plots of the individual data points on a principal component analysis projection colored according to a k-means clustering. The borders of the colored areas visualize the cluster separation. The right panels **D, H and L** show Silhouette plots for the three clusters. Positive values indicate that the sample is within a cluster while negative values indicate that those samples might have been assigned to the wrong cluster because they are closer to neighboring than to their own cluster. The figure has been created using the R software package (version 4.1.2 for Linux; https://CRAN.R-project.org/) and the R packages “ggplot2” (https://cran.r-project.org/package=ggplot2), and “FactoMineR“ (https://cran.r-project.org/package=FactoMineR). The colors were selected from the “colorblind_pal” palette provided with the R library “ggthemes” (https://cran.r-project.org/package=ggthemes).

Figure 1


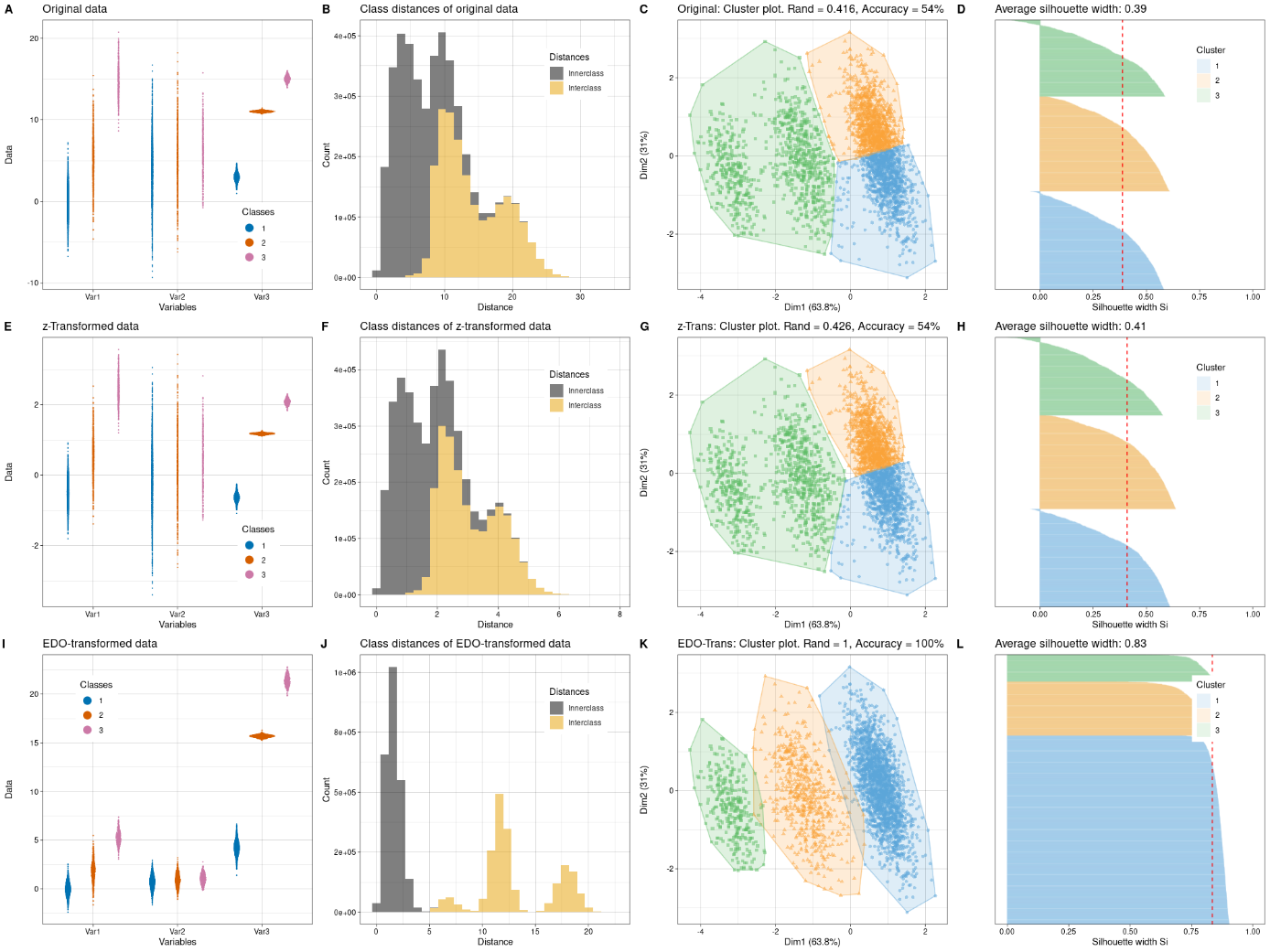


## Average linkage for hierarchical clustering

Supplementary Figures 2 – 5, equivalent to Figures 3 – 6 of the main report, with the exception that average linkage instead of Ward’s linkage was used for clustering. For details description, please see the legends of the mentioned figures of the main report.

Figure 2


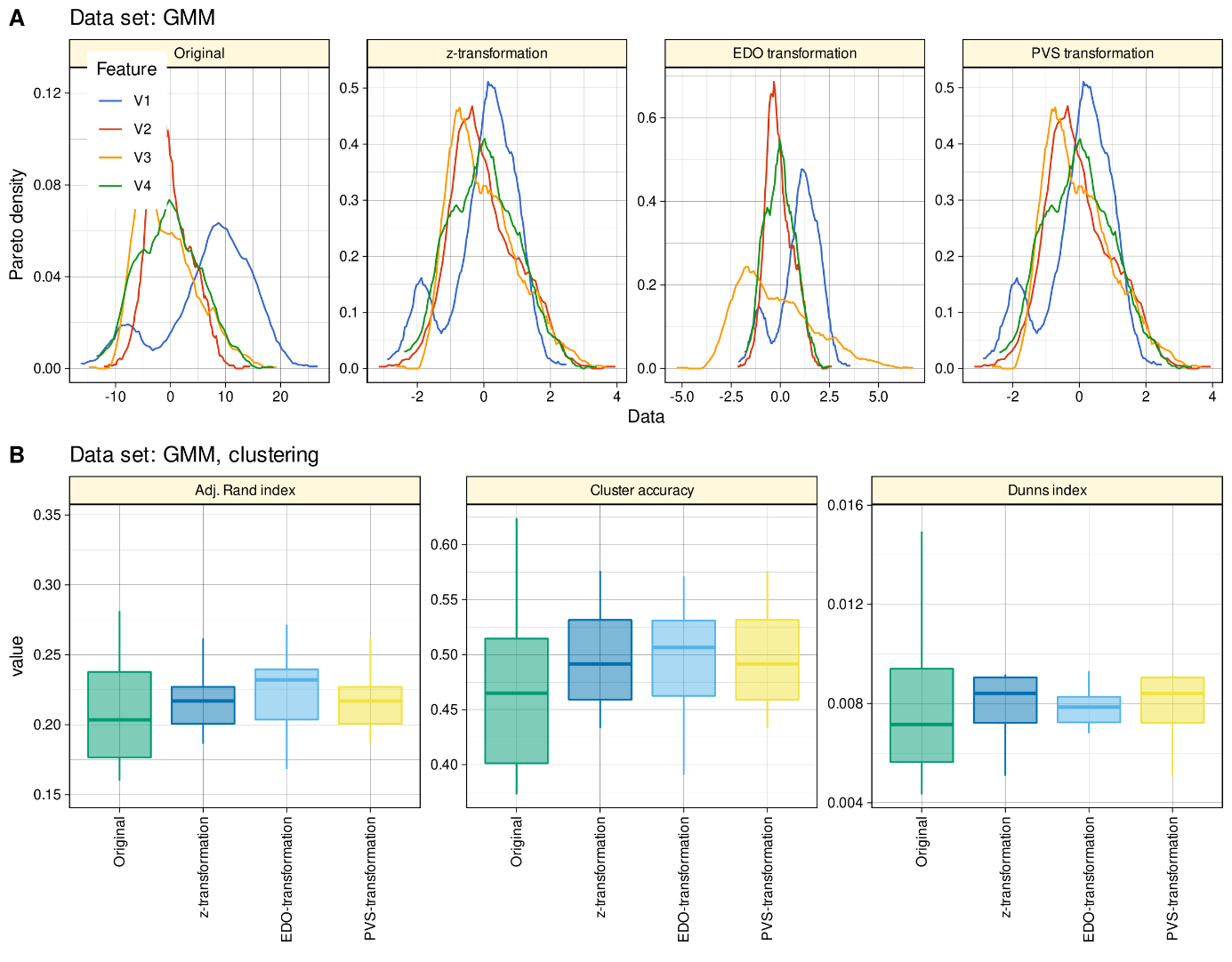


Figure 3


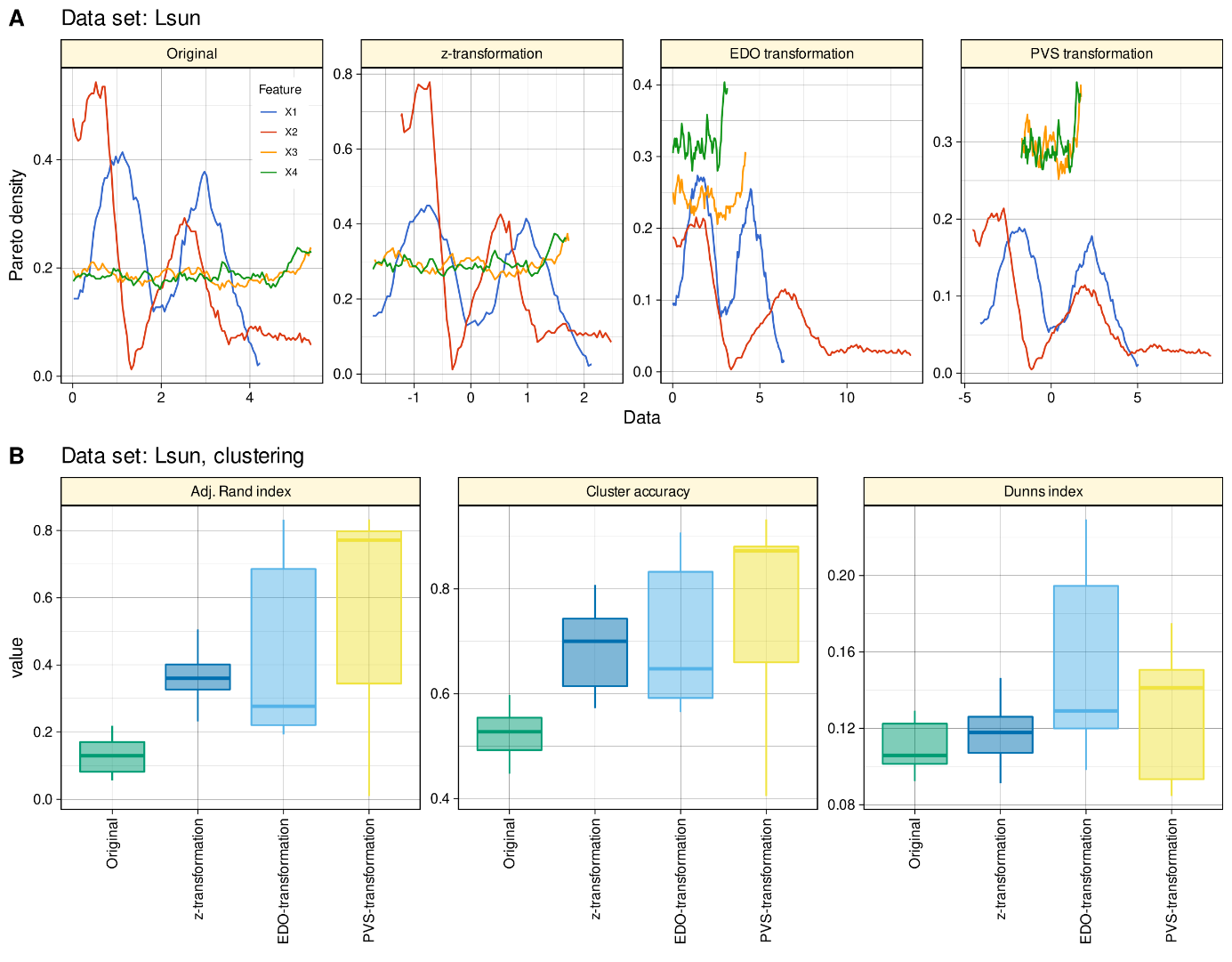


Figure 4


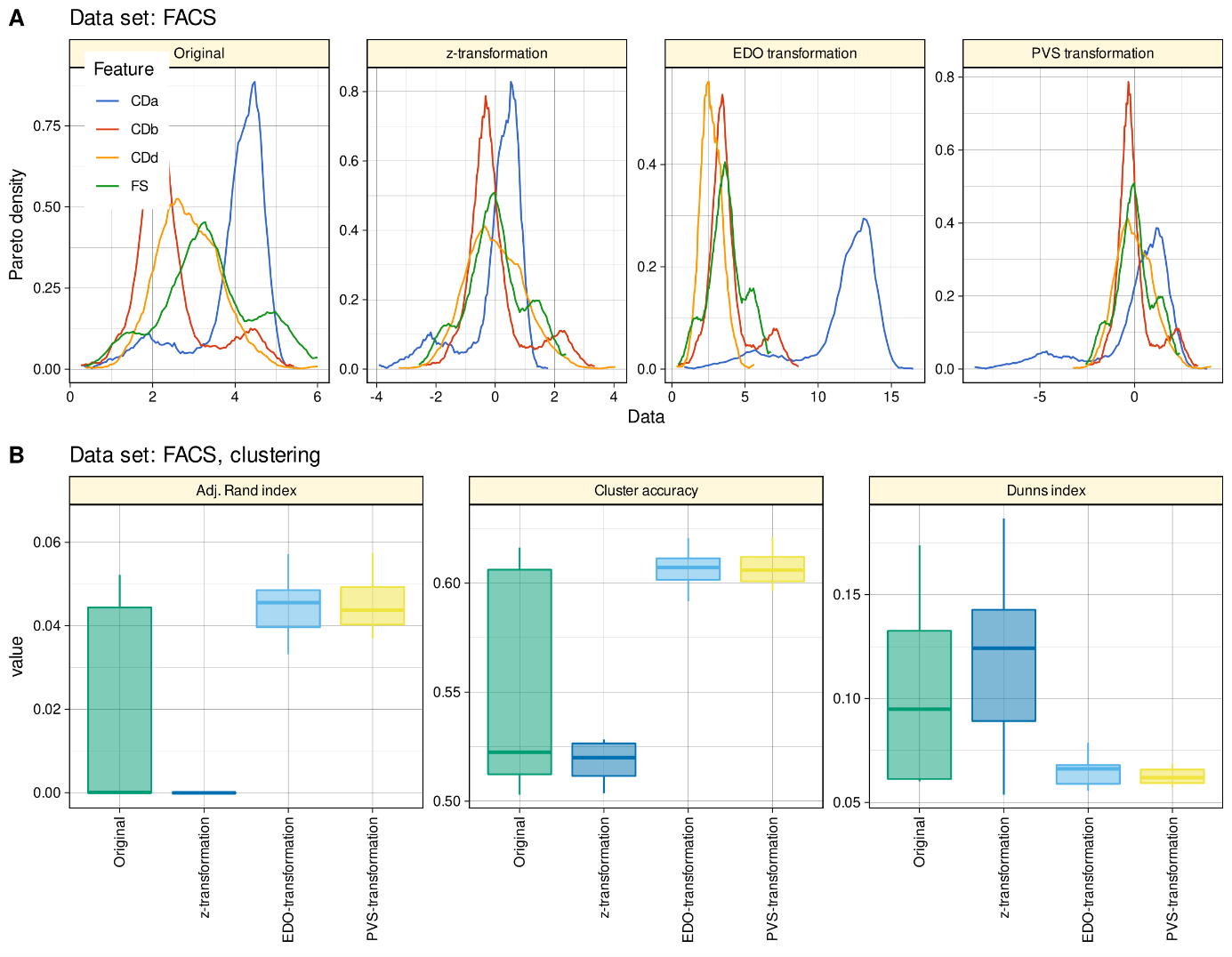


Figure 5


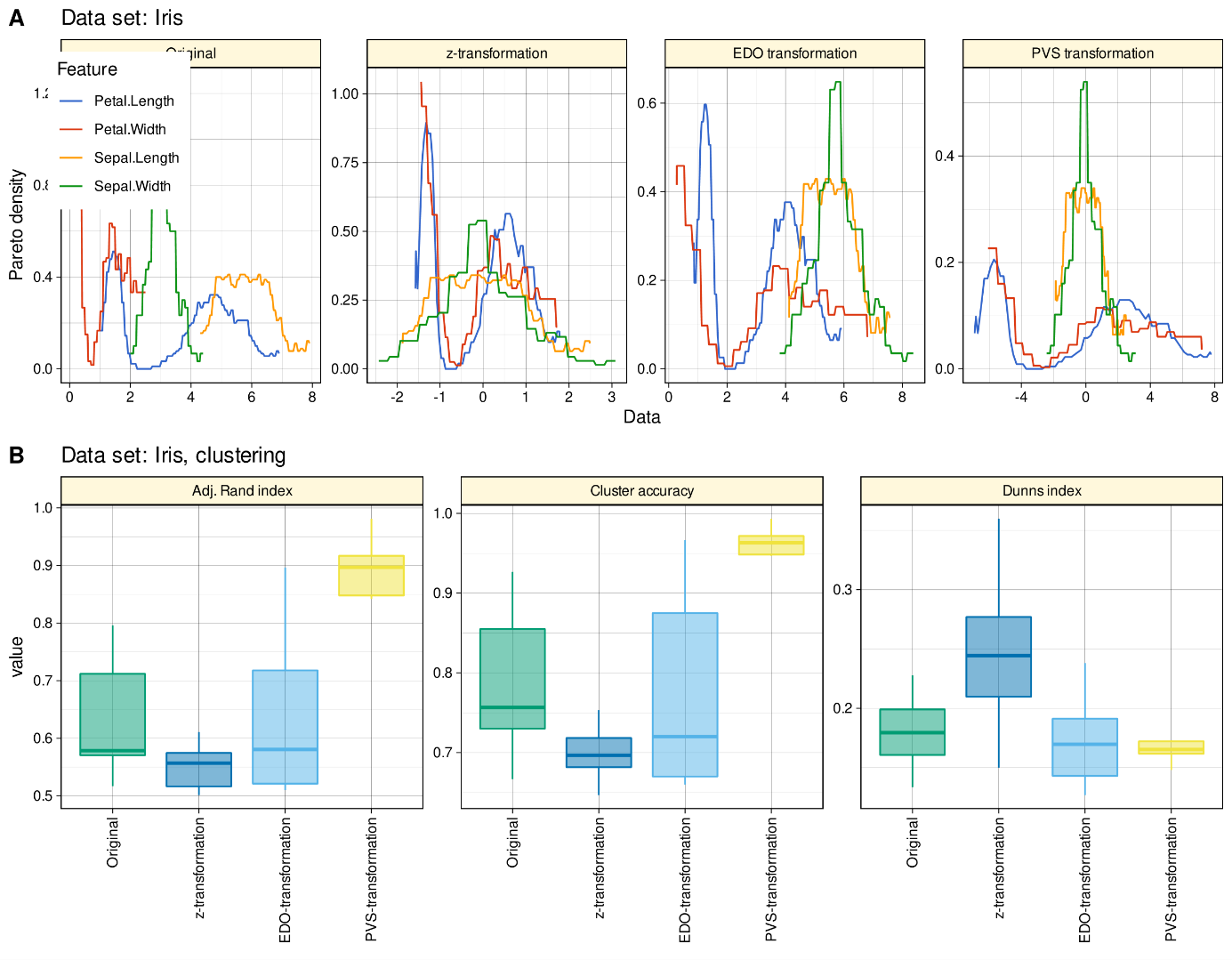


## Complete linkage for hierarchical clustering

Supplementary Figures 6 – 9, equivalent to Figures 3 – 6 of the main report, with the exception that complete linkage instead of Ward’s linkage was used for clustering. For details description, please see the legends of the mentioned figures of the main report.

Figure 6


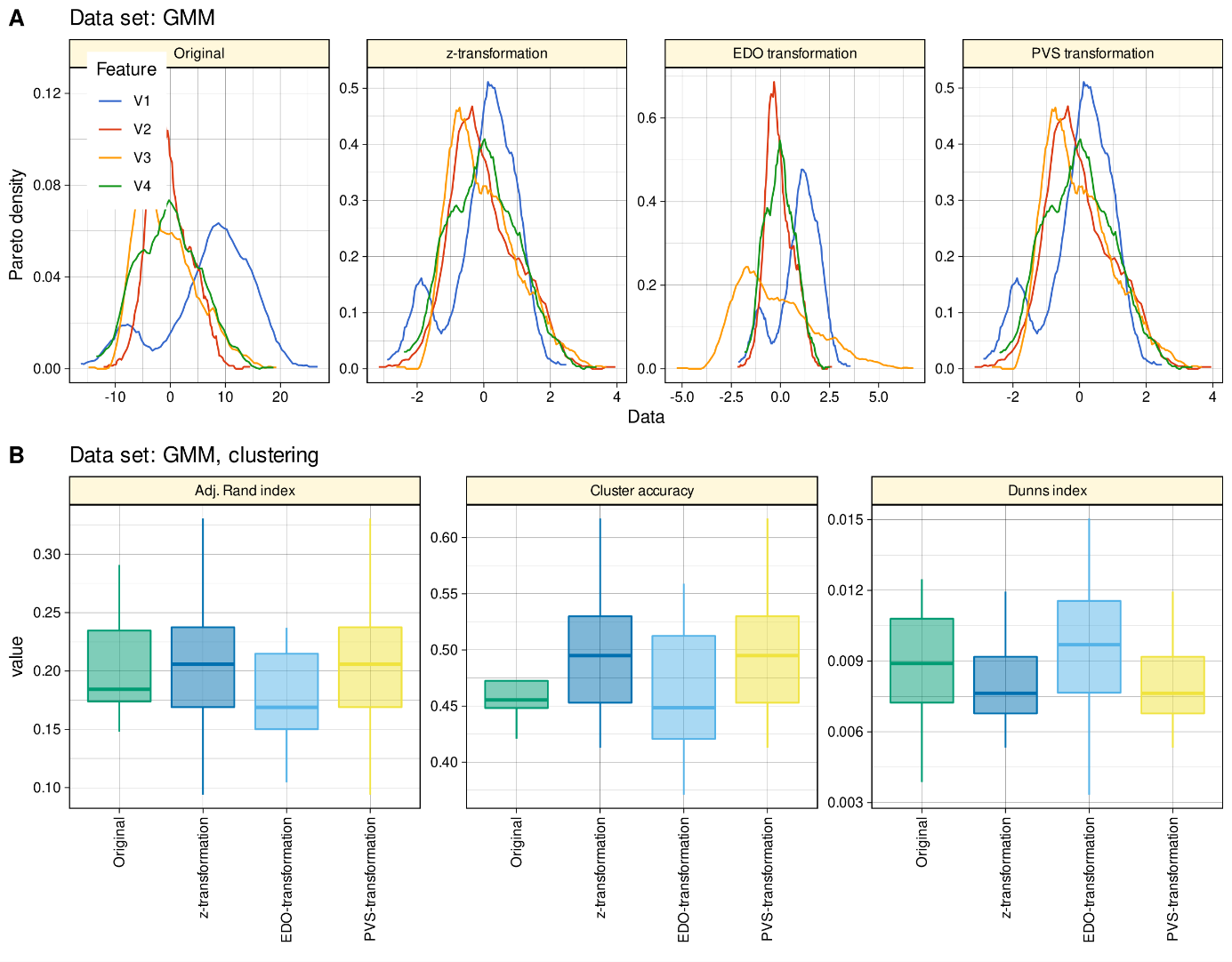


Figure 7


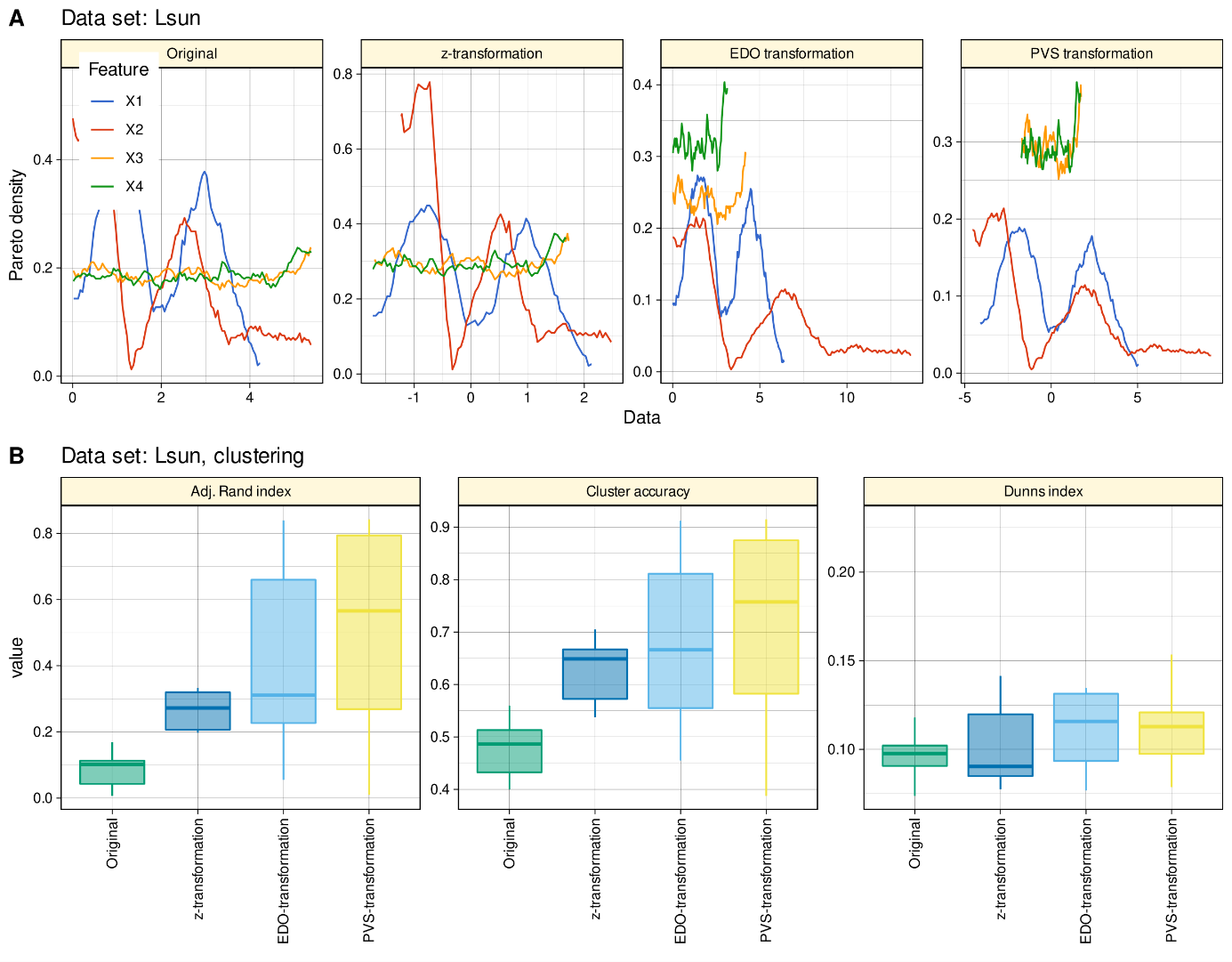


Figure 8


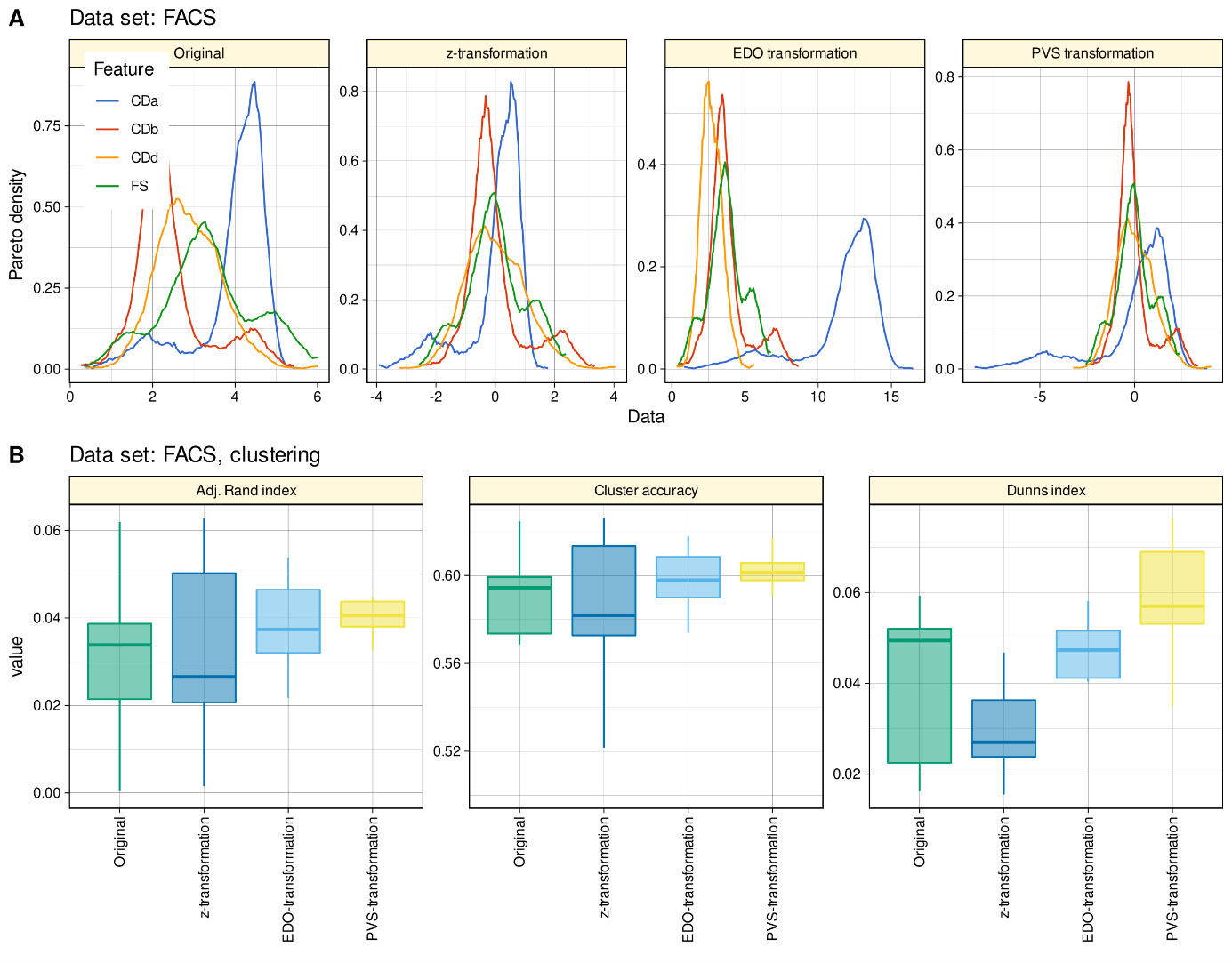


Figure 9


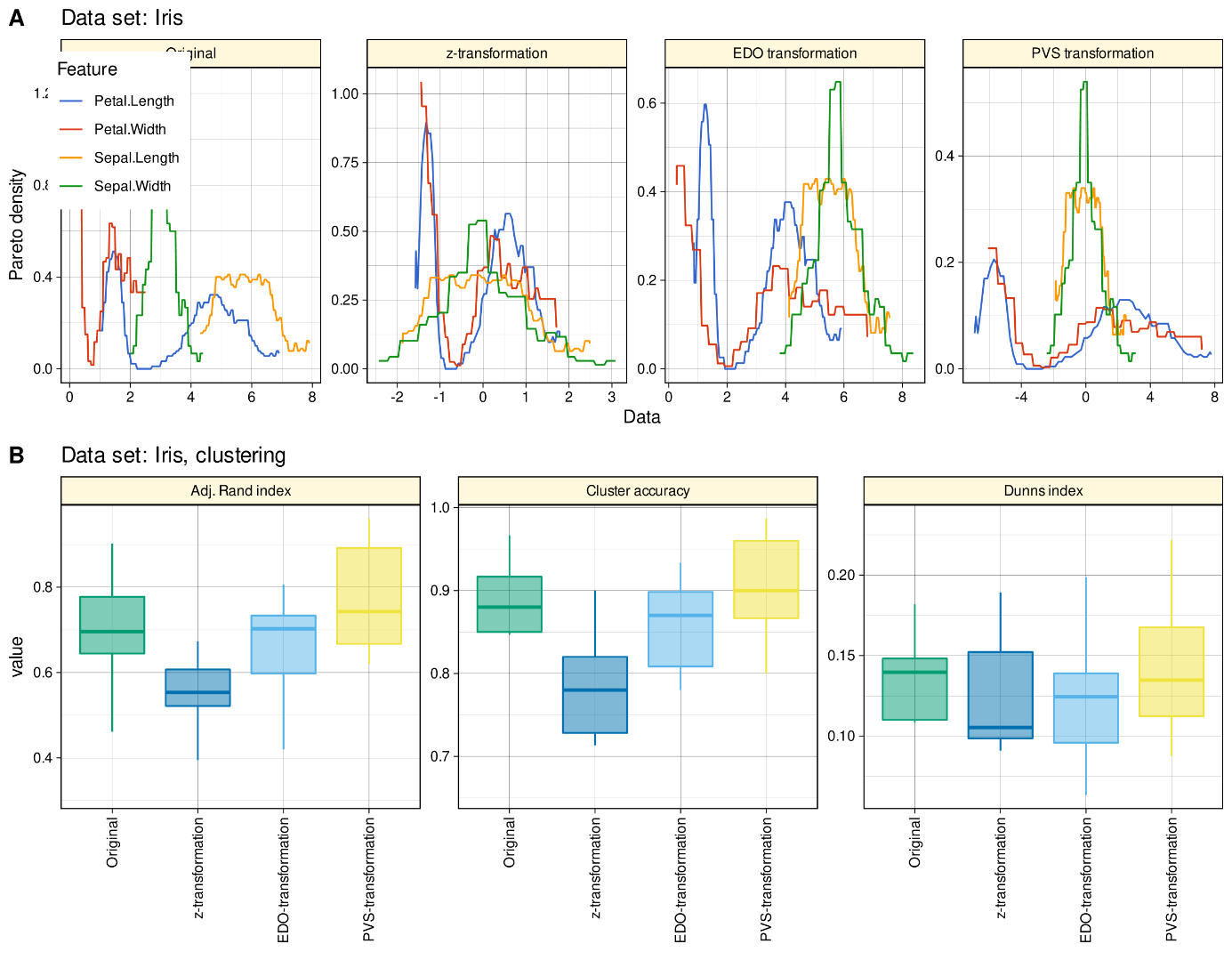

Supplement: Supplementary file 1 — Additional file 1. Supplemental figures, showing the results of the proof-of-concept study using PAM clustering instead of k-means, and results of the three experiments with the data sets of Gaussian mixtures, Iris flowers and FACS data, using average or complete linkage instead of Ward’s linkage. [file 12859_2022_4769_MOESM1_ESM.docx]
